# The modular systems biology approach to investigate the control of apoptosis in Alzheimer's disease neurodegeneration

**DOI:** 10.1186/1471-2202-7-S1-S2

**Published:** 2006-10-30

**Authors:** Lilia Alberghina, Anna Maria Colangelo

**Affiliations:** 1 Department of Biotechnology and Biosciences, Laboratory of Neuroscience "R. Levi-Montalcini", University of Milano-Bicocca, 20126 Milan, Italy

## Abstract

Apoptosis is a programmed cell death that plays a critical role during the development of the nervous system and in many chronic neurodegenerative diseases, including Alzheimer's disease (AD). This pathology, characterized by a progressive degeneration of cholinergic function resulting in a remarkable cognitive decline, is the most common form of dementia with high social and economic impact. Current therapies of AD are only symptomatic, therefore the need to elucidate the mechanisms underlying the onset and progression of the disease is surely needed in order to develop effective pharmacological therapies. Because of its pivotal role in neuronal cell death, apoptosis has been considered one of the most appealing therapeutic targets, however, due to the complexity of the molecular mechanisms involving the various triggering events and the many signaling cascades leading to cell death, a comprehensive understanding of this process is still lacking.

Modular systems biology is a very effective strategy in organizing information about complex biological processes and deriving modular and mathematical models that greatly simplify the identification of key steps of a given process.

This review aims at describing the main steps underlying the strategy of modular systems biology and briefly summarizes how this approach has been successfully applied for cell cycle studies. Moreover, after giving an overview of the many molecular mechanisms underlying apoptosis in AD, we present both a modular and a molecular model of neuronal apoptosis that suggest new insights on neuroprotection for this disease.

## 1. Introduction: the modular systems biology approach

Systems biology aims at describing biological processes as interactions of molecular components structured by regulatory wirings, whose dynamics in various conditions might be predicted by using mathematical models and computer simulations. The goal is to achieve the ability to integrate in a comprehensive model of pathways and networks the huge amount of data coming from post-genomic analysis in order to gain a better understanding of biological processes and a presently unattainable predictive ability.

For relatively simple processes, such as glycolysis, the structure of the pathway is quite well defined and allows us to readily construct the corresponding model. Neverthless, it has to be underlined that also in this relatively well known situation the systems biology approach has been able to identify a previously underestimated rate limiting step [1].

For other more complex processes, such as cell cycle, apoptosis, differentiation, transformation, we lack both a complete molecular understanding and the logic of their regulatory wirings. In order to develop a systems biology approach able to foster the understanding of complex bioprocesses, our laboratory has developed a modular systems biology approach and tested its validity in cell cycle studies.

The notion is by now largely accepted that cellular processes are carried out by modules, subsystems of interacting molecules (DNA, RNA, proteins and metabolites) that perform a given function in a way largely independent from the context [2]. The various modules are linked by governing interactions that follow general design principles well known in engineering, such as positive and negative feedbacks, switch, thresholds, amplification, error correction, etc. [3].

The modular systems biology approach, which is extensively discussed in ref. [4], starts with a global functional analysis of the process in order to identify both the known players and the main control functions active in the process. Then a modular blueprint of the process is constructed by gathering all the information previously collected. This low resolution blueprint is then used to derive a mathematical model that, tested by simulation, will show whether it is able to capture the essential features of the process. In the negative case, one has to go back to the drawing board and reconsider the blueprint structure; in the positive case, one can move on to identify the molecular components of each module following the so called **4M **Strategy: **M**ining of literature and data banks; **M**anipulation by genetic means and by environmental changes of the module structure and function; **M**easurements of all putative regulatory components supposed to be active in the module (i.e. estimation of their concentration, localization, state of activation, time-course changes, etc.); **M**odeling and simulation: construction of a model at higher resolution than the previous one, in which the putative regulatory components of the module are linked by a specific flow of events. The simulation of this model will be compared with experimental results, in order to check its validity. It starts, therefore, an iteractive process of modeling, computer simulation, comparison with experimental data, new hypothesis-driven experiments, computer simulations, predictions that are going to be experimentally tested, which is the characteristic of the systems biology approach [5].

**Figure 1:**
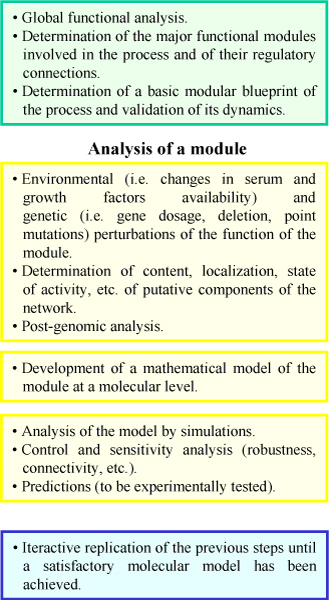
Scheme of the iterative roadmap of computational and experimental approaches applied in modular systems biology.

In this review we are going to apply the modular systems biology approach to give structure to the information available about the role of apoptosis in neurodegenerative diseases. Moreover, we construct the blueprint of neuronal apoptosis and present a tentative molecular model of the mechanisms underlying neuronal apoptosis. The roadmap describing the process is briefly reported in Figure 1.

In order to explain how the modular systems biology approach works in the definition of a molecular model of complex biological processes, we briefly summarize how this approach has been successfully used in our laboratory for cell cycle studies.

## 2. The modular systems biology approach for cell cycle studies

Cell cycle requires the coordination of different processes: mass accumulation, DNA replication and faithful partitioning of genetic material. In the budding yeast *Saccharomyces cerevisiae*, the most relevant model organism for cell cycle studies, the coordination of mass accumulation with cell cycle progression relies on a cell sizer mechanism that allows DNA replication to start only when cells have reached a critical cell size, called Ps. Although this key regulatory control of cell cycle has been known for almost three decades [6-8], even recent cell cycle models [9,10] do not offer satisfactory molecular explanations for the setting of Ps. Besides, it is well known that external and metabolic signals are able to control the setting of the critical cell size [11], but we still ignore the detailed mechanisms that link signal transduction pathways to the cell cycle core molecular machine.

### 2.1. Defining the cell sizer regulatory network controlling entrance into S phase in yeast

In order to define the molecular mechanism underlying the cell sizer, the first step has been the drawing of a blueprint of the cell cycle [12] that states that the cell sizer is based on a threshold mechanism involving a growth-sensitive cyclin (Cln3) and an inhibitor of cyclin-dependent kinase (Cki), Far1, previously known to be involved in the arrest of the cell cycle in G1 in response to mating factors [13-15]. After the execution of the growth-sensitive threshold, a cascade of cyclin-dependent-kinases takes place, that controls the sequential progression of the cycle, terminated by an End function, that comprises events from the onset of mitosis to cell division and whose progression may be regulated by stress [12,16].

The second step has been the gathering of experimental evidence about the involvement of Far1 in cell cycle control: an increased level of Far1 increases cell size, whereas FAR1Δ cells initiate bud emergence and DNA replication at a smaller size than wild type. Besides Cln3Δ, FAR1Δ and strains overexpressing Far1 do not show a drop in budding (a marker of the entrance into S phase) during an ethanol-glucose shift-up as wild type does. The activation of Cln3·Cdk1, following the overcoming of the Cln3/Far1 threshold, activates SBF- and MBF-dependent transcription. A second threshold given by Clb5,6·Cdk1/Sic1 is required, together with the first one, to adjust the Ps value to carbon sources availability (i.e. low Ps in poor/ethanol media, high Ps in rich/glucose media) [17].

A mathematical model accounting for the network identified so far has been implemented and shown to capture basic features of the G1 to S transition, such as the role of Cln3 and Far1 dosage on the setting of Ps [4].

The third step has focused more on the role of Sic1, another Cki, in the control of the entrance into S phase, focusing on the modulation by carbon source of the subcellular localization of Sic1 [18]. Sic1 and the cyclins Clb5 and Clb6 are the major regulators of the Cdk1 kinase activity required for the onset of DNA replication [19]. The complex Clb5,6·Cdk1/Sic1, formed in G1 cells when Clb5,6 are synthesized due to MBF-mediated transcription, is activated by proteolysis of Sic1, primed by Cln1,2·Cdk1 mediated phosphorylation on multiple sites and dependent on SCF complex [20].

The carbon source modulation of the setting of Ps mediated by Sic1 is dependent upon the fact that carbon source affects both the overall level and the subcellular localization of Sic1, that requires a newly characterized NLS to be imported in the nucleus. Besides, it has been shown that Sic1 facilitates nuclear accumulation of Clb5, thus playing a hitherto unrecognized positive role in promoting the G1 to S transition [18]. The kinetic parameters of the binding of Sic1 to cyclin/Cdk complex have been estimated, and evidence has been obtained *in vitro *showing that the strength of binding may be modulated by Sic1 protein phosphorylation [21].

It may be interesting to recall that Sic1 has been shown to be a functional homologue of the mammalian p27^Kip1 ^with a conserved inhibitory domain [22]. Also p27^Kip1^, together with p21^Cip1^, has been shown to promote nuclear import of cognate cyclin D1/Cdk4, therefore the import of cyclins, that do not have obvious nuclear import signals, like cyclin D in mammals and Clb5 and Clb6 in yeast, appear to be imported into the nucleus piggy-backed on cargos (the complex cyclin/Cdk/Cki) that contains a protein (the Cki) with a NLS [23,24].

The fourth and last step, so far, has been to derive a mathematical model of the complex nucleus/cytoplasmic network, derived from the previous experimental findings integrated with the literature data, and to analyze various aspects of it by simulation [Barberis et al., 2006, submitted for publication]. Besides accounting for the behaviour of many known cell cycle mutants, the model shows that the critical cell size depends on the structure of the network and, in a more sensitive way, on few parameter values. The results of the simulations shed new light on the previously reported involvement of ribosome biogenesis on the setting of Ps [25-27].

## 3. Why modular systems biology approach for neurodegenerative diseases?

Taken together, the above mentioned results offer a strong support to the notion that the modular systems biology approach is very effective in fostering the identification of networks controlling complex bioprocesses and, therefore, it could be useful for a better understanding of neuronal apoptosis. Recently, mathematical models have been developed for CD95- and cytokine-induced apoptosis in mammalian cells [28,29], as well for Ca^++ ^signaling at dendritic spines and neurotransmitter receptor complexes underlying synaptic plasticity [30,31]. This review discusses the use of the modular systems biology approach to investigate apoptosis in neurodegenerative disorders and, in particular, Alzheimer's disease (AD). The basic idea is that structuring the apoptotic process in modules will allow us to better understand the regulatory wiring of the process.

As previously discussed, the first step requires the collection and organization of the information available about the process being considered. Therefore, the following paragraphs are going to gather current knowledge about functional analysis of apoptosis in AD neurodegeneration in order to derive the structure in modules of the blueprint, followed by an analysis of the key molecular players, in order to structure a tentative molecular model of neuronal apoptosis to be proposed for experimental validation.

## 4. Global functional analysis of apoptosis in AD

Neurodegenerative diseases are characterized by dysfunction and death of specific neuronal populations and, in many instances, by the presence of intracellular and extracellular aggregates of otherwise soluble proteins, a common feature in several disorders of the central nervous system (CNS), such as AD, Parkinson's (PD) and Huntington's diseases (HD) and Amyotrophic Lateral Sclerosis (ALS) [32-34].

Besides the genetic component of different molecular and cellular alterations, neuronal degeneration is contributed by the intervention of various factors and biochemical events that are relevant also to acute neuropathological conditions and including metabolic and oxidative stress, disruption of Ca^++ ^homeostasis and excitotoxicity, as well neurotrophic factor deprivation or traumatic injury [32,33,35-37]. For instance, energy depletion, excitotoxicity and oxidative stress are the major pathological mechanisms leading to neuronal death during ischemic stroke after oxygen and glucose deprivation due to transient or permanent reduction of the cerebral blood flow [38]. On the other hand, many biochemical mechanisms leading to cell death are shared by several neuropathological conditions. For example, excitotoxicity due to overactivation of glutamate receptors represents a final common pathway for both acute neurological disorders, such as stroke, trauma and epileptic seizures, and chronic neurodegenerations like AD [35,39].

Regardless of which is the initial trigger and the neuronal population affected, neuronal death can occur through mechanisms involving necrosis or apoptosis, depending on the intensity and duration of the insult, both processes involving mitochondrial function [40,41]. Necrosis is a rapid form of death resulting from an acute toxic event that leads to complete failure of mitochondrial function and cellular homeostasis. Apoptosis is, instead, a slow and active process triggered by less severe insults and represents a mechanism for neurons to delay death, while activating various neuroprotective programs involving molecules and genes with anti-apoptotic function [42]. As discussed below, in this process, that is characterized by cell shrinkage, plasma membrane blebbing, nuclear chromatin condensation, DNA fragmentation and increase of a number of apoptotic markers, mitochondria play a pivotal role in cell decision, which is supposed to depend on a balance between apoptotic and anti-apoptotic signals. The initial trigger, in fact, induces a state of increased vulnerability to other toxic events and/or synergize with other apoptotic cascades, thus causing a persistent imbalance of neuronal homeostasis, mitochondrial dysfunction and neuronal death.

Increasing evidence indicates that apoptotic pathways occur in most neurological disorders. Besides the role of apoptosis in excitotoxicity and traumatic injury, there is evidence for apoptotic cell death in the penumbral region of ischemic brains [41,43,44]. Furthermore, apoptosis is currently regarded as the main form of death during neurodegenerative disorders like AD, PD, HD and ALS, as supported also by a number of studies in patients [45-47].

AD is a progressive neurodegeneration characterized by a disruption of synaptic function leading to a remarkable cognitive decline, including memory impairment and behavioural changes. The pathological hallmarks of AD include the presence of aggregates of amyloid-β peptide (Aβ) in neuritic plaques and intracellular neurofibrillary tangles (NFTs) of hyperphosphorylated tau (PHFtau), together with loss of neurons and synapses in the neocortex, hippocampus and other subcortical regions of the brain. These features are present both in sporadic AD and in early-onset familial form (FAD) which has been associated with genetic mutations of the β-amyloid precursor protein (APP) and the presenilin-1/2 (PS1/2) genes [34 and refs therein].

Due to the strong social impact of AD, a large number of studies have focused on the pathological mechanisms underlying this debilitating disease that represents the most common form of dementia.

Neurochemical analyses indicate that neurotransmitter deficit, neuronal atrophy and synaptic dysfuction are early events in AD, as they occur also in subjects with Mild Cognitive Impairment (MCI), a high-risk pre-dementia state of the disease. Decrease of cholinergic markers and loss of basal forebrain cholinergic neurons has been observed in the brain of AD patients and senile dementia [48], and a strong correlation has been found between the degree of cognitive deficit and the decrease of synaptophysin immunoreactivity [49].

Brain imaging, electrophysiological and neuropsychological studies have shown that the degree of synaptic loss is correlated to levels of soluble oligomers of Aβ and occurs even before the appearance of Aβ plaques and NFT formation [50-54]. However, a reduction of neurotrophic support, in particular Nerve Growth Factor (NGF) [55-57] and Brain-Derived Neurotrophic Factor (BDNF) [58], is also believed to play a role in neuronal dysfunction, as discussed below.

Current therapies of AD are only symptomatic and include treatments with acetyl-cholinesterase inhibitors to improve cognitive function. However, effective therapies may require the development of pharmacological strategies that enable to modify and reverse the many biochemical alterations involved in the onset and progression of the disease.

Given its well established role in AD degeneration, apoptosis has lately gained much attention as a potential pharmacological target. However, although many of the apoptotic pathways have been extensively elucidated in terms of signaling [59-63] and differential gene expression [64,65], a comprehensive understanding of the apoptotic process in AD is still missing, due to the complex cross-talk between many different signaling cascades that are activated in a dynamic and coordinated fashion. Besides Aβ toxicity and deficit of neurotrophic support, neuronal death is triggered by a huge number of signals and biochemical mechanisms including microtubule transport impairment, decreased glucose metabolism, excitotoxicity, oxidative stress, mitochondrial dysfunction and inflammatory processes [37,59,61,66,67].

In order to apply the modular systems biology approach to apoptosis in AD, we will next review both the biochemical basis of the physio-pathological events underlying the two current AD hypothesis and the molecular mechanisms through which a number of diverse neurotoxic insults in AD may actually converge to convey apoptotic signals. Then, we will attempt for the first time to structure them in a molecular/modular model.

### 4.1 The *"Neurotrophic Factor Hypothesis" *and AD neurodegeneration

The "*Neurotrophic Factor Hypothesis" *[68,69] is linked to the discovery of NGF by R. Levi-Montalcini and the first observations of cell death for neurons deprived of their target of innervation [70,71]. The neurotrophic activity of target-derived NGF was then followed by the identification of other molecules including BDNF, Neurotrophin-3 (NT-3) and Neurotrophin-4/5 (NT-4/5), with neurotrophic activity on different neuronal populations. All neurotrophins share similar sequences and structures along with variable domains that determine the specificity of their biological activity resulting from the interaction with their cognate tyrosine kinase (Trk) receptor [[72] and refs. therein].

Thus, the phenomenon of *apoptosis*, that is central to the *Neurotrophic Factor Hypothesis*, was first observed in the context of the discovery of NGF and its role on differentiation and survival of sympathetic and sensory neurons of the peripheral nervous system [70,71]. During development, neurotrophins regulate neuronal survival and differentiation, and determine the *pattern *of innervation and the expression of proteins that are crucial to a specific neuronal phenotype, like neurotransmitters and neuropeptides, neurotransmitter receptors and ion channels. According to the neurotrophic hypothesis, the correct pattern of innervation is dependent upon competition of developing neurons for a limited supply of growth factors secreted by target tissues, thus ensuring a balance between the number of neurons and the size of the innervated target [68,69].

Neurotrophins activity, however, is essential throughout adult life and aging, where they play a key role in the regulation of neuronal function and synaptic plasticity, as well in neuroprotection and repair [73]. In this context, neurotrophins function can be revelant to the development of neuronal dysfunction and degeneration.

The potential relevance of NGF to neuronal loss in AD is based on a large body of evidence, including the existence of a strong correlation between NGF expression in neocortex and hippocampus and expression of TrkA receptor and choline acetyltransferase (ChAT) activity in the basal forebrain, both during development [74-76] and in the adult [77,78]. Cholinergic neurons atrophy and memory deficit in aged rat and in primates could be prevented by intraventricular infusions of NGF [79-82], while it is induced by intraventricular administration of anti-NGF antibodies [55] or disruption of the NGF gene [56]. Moreover, there is evidence for reduced or defective retrograde transport of NGF in aged rats [83], while increased cortical and hippocampal NGF levels with reduction of NGF in the nucleus basalis have been found in AD patients [36].

Neverthless, it was shown that excitotoxic lesion of target neurons does not induce death of basal forebrain cholinergic neurons, but cell shrinkage and decreased ChAT activity, suggesting that in the adult these neurons are no longer dependent on NGF for acute survival but only for maintenance of their cholinergic phenotype that is crucial to the modulation of cortical and hippocampal synaptic plasticity and learning and memory processes [84].

Recently, the potential role of NGF in AD degeneration has been supported by the AD model of anti-NGF transgenic mice [57,85]. The conditional expression of antibodies in the adult induces cholinergic deficit and the appearance of structural changes (amyloid plaques, PHFtau and NFTs) resembling those found in human AD, thus establishing a strong correlation between NGF deprivation and cholinergic dysfunction. Moreover, the cholinergic deficits of these mice could be rescued by intranasal administration of NGF [86].

Besides NGF, it must be acknowledged also the role of BDNF in AD, given the well established role of this neurotrophin in synaptic plasticity of the hippocampus and the requirement for BDNF in long-term potentiation (LTP) during learning and memory processes [87]. The potential role of BDNF in AD is supported by several studies showing a reduction of BDNF mRNA and protein levels in the cortex and hippocampus of MCI and severe AD patients [58,88]. As this reduction also occurs at early stage of the disease, it clearly demonstrates a role for decreased BDNF in the cognitive impairment in AD, that even precedes the reduction of ChAT activity [89].

An intriguing issue about the relevance of neurotrophins in the onset and/or progression of AD is the recent finding that the neurotrophin precursor proNGF appears to be the major form of NGF in the brain and that proNGF levels are increased in the parietal cortex of AD patients, even at early stages of the disease and in subjects with MCI [90]. ProNGF levels were also found to be enhanced after CNS injury, together with upregulation of the p75 neurotrophin receptor and increased p75-mediated apoptosis [91]. Enhanced p75 levels are known to occur in various injuries of the nervous system, as shown by both *in situ *hybridization and immunohistochemistry analyses [92-94]. In AD patients, increased p75 expression [95] is accompanied by a 50% reduction in TrkA levels [96], thus providing a challenging interpretation for decreased NGF survival signaling and increased p75-induced apoptosis.

It is well known that levels of expression of NGF and its receptors TrkA and p75 determine a balance between survival and apoptosis responses, the latter induced by NGF in the absence of TrkA receptor or upon alteration of the ratio between the two receptors in cells that express both TrkA and p75 [97]. The discovery that NGF may be secreted also as proNGF and that this unprocessed form can induce neuronal death through preferential interaction with the p75 receptor suggest that NGF may play a role in neuronal survival also in the adult where life-death decision would be also dependent upon the ratio between secreted mature and proNGF [98,99].

Recently, also BDNF was found to be secreted as proBDNF by cultured neurons and shown to induce apoptosis of sympathetic neurons [100]. However, proNGF and proBDNF do not induce apoptosis in all p75-expressing neurons, as their pro-apoptotic activity appears to depend on co-expression of sortilin, a member of the newly discovered family of Vps10p-domain receptors [100,101]. Sortilin is expressed both during embryogenesis and in the adult, in neurons and non neuronal cells of different brain areas including cortex, hippocampus, medial and lateral septum and amygdala [102].

These findings add a further level of complexity to that already given by the large number of different biological activities triggered by NGF upon activation of TrkA and p75 receptors, as well as that resulting from the complex cross-talk between the molecular intermediates of the signaling cascades activated individually by each receptor. Moreover, new questions arise as to whether proneurotrophins may have their own functional role under physiological conditions, or whether they result from postranslational alterations of neurotrophin synthesis during brain damage. Interestingly, plasmin levels are reduced in the brain of AD patients [103], although plasmin is a proteinase that cleaves proNGF and proBDNF *in vitro*, but its activity *in vivo *is still uncertain. Moreover, a recent report showed a role of proBDNF-p75 in the enhancement of long-term depolarization (LTD) mediated by upregulation of the NR2B subunit of the N-methyl-D-aspartate (NMDA) receptors [104].

The neurotrophic hypothesis of AD has led to some phase I clinical trials. One study was based on intraventricular administration of NGF in three AD patients [105], while the last trial involved a rhNGF gene therapy approach that resulted in some beneficial effect on cognitive function [106].

Due to the invasiveness of these strategies, together with the poor NGF permeability across the blood-brain barrier, many studies have also focused on pharmacological regulation of endogenous NGF expression [107]. Recently, a phase II clinical trial has been carried out by administration of a γ-aminobutyric acid (GABA) receptor antagonist in 110 MCI patients. [108]. The drug increased NGF and BDNF mRNA and protein levels in the rat cortex and hippocampus, and improved cognitive function in the patients. A second trial in AD patients is still in progress.

It should be emphasized that a NGF-based therapy of AD may not be sufficient to fully reverse the AD pathology. However, due to its role in neuroprotection and repair, it is expected that NGF may provide a useful tool to ameliorate the symptoms and slow down the progression of the disease, yielding anyhow a relevant accomplishment as compared to the present therapeutic interventions. A more effective AD therapy might be better accomplished by targeting also other specific mechanisms of the disease cascade, such as apoptosis.

### 4.2 AD and the *Amyloid hypothesis*

As mentioned, a clear role for increased production and accumulation of soluble Aβ in the pathogenesis of AD has been supported by a large number of studies [34,50-54].

Generation of neurotoxic species of Aβ results from an abnormal metabolism of APP, a membrane protein localized at the presynaptic terminals [[109] and refs therein]. A non-amyloidogenic pathway involves cleavage of APP by α-secretase and production of a soluble secreted form of APP (sAPPα), which has several biological activities including cell survival, neurite outgrowth and protection against excitotoxicity [110]. Production of amyloidogenic Aβ occurs, instead, through sequential cleavage of APP at the N-terminus by β-secretase and in the transmembrane domain by γ-secretase, a multimeric complex whose proteolytic activity is regulated by binding of PS1/2 at its catalitic site. Cleavage sites of γ-secretase are located at positions 40 and 42, thus generating Aβ 40 or Aβ 42, respectively [109].

Although Aβ 42 is constitutively produced in the brain, mutations in the APP or PS1/2 genes (which alter the cleavage specificity of γ-secretase) lead to increased production and accumulation of Aβ 42, that is neurotoxic even before its aggregation in Aβ fibrils [111]. In fact, soluble Aβ (including Aβ oligomers) can compromise basal synaptic transmission and LTP in the hippocampus even in the absence of Aβ deposits [53,54]. Synaptic activity and learning are impaired also in APP transgenic mice [112]. Additional studies in cultured cortical neurons have shown that sublethal concentrations of Aβ decrease the activity of cyclic AMP-response element binding protein (CREB) and downstream gene expression, such as the activation of the BDNF promoter [113]. CREB is also a transcription factor for the NGF promoter [114]. Sublethal doses of Aβ also interferes with BDNF-mediated pathways involved in protection of cortical neurons from apoptosis [115]. Given the role of BDNF in LTP during learning and memory [87], these data establish a strong link between Aβ, trophic support and learning-memory impairment in AD degeneration.

The correlation between Aβ and neurotrophins function is supported by several studies showing that the amyloidogenic pathway seems to be favored by a failure of TrkA-mediated NGF signaling and dysfunction of cholinergic neurons [109], where NGF regulates ChAT activity, choline uptake, acetylcholine synthesis and release [116]. In PC12 cells, APP transcription and secretion have been found to be regulated by NGF and its receptors TrkA and p75: TrkA activation increases APP processing and secretion, while p75 signaling increases APP transcription [117]. Recently, these data have been confirmed by *in vivo *studies showing that accumulation of Aβ in aging might depend on a switch of TrkA-p75 expression and that p75 enhances Aβ production in a NGF-dependent manner by a ceramide-mediated mechanism [118].

Likewise, the APP secretory pathway, which generates nonamyloidogenic sAPPα, is increased by activation of the phosphoinositides (IP3)/Protein kinase C (PKC) signaling pathways through stimulation of muscarinic acetylcholine receptor (mAChR)/G protein coupled receptors (GPCR) both *in vitro *and *in vivo *[119,120]. Cortical and hippocampal M1/M3 mAChR signaling is impaired in AD, although the number of receptors is unaltered [121], suggesting that an impaired mAChR signaling may favor the amyloidogenic pathway of APP. On the other hand, a reciprocal correlation also exists, since Aβ has been shown to decrease acetylcholine synthesis and impair muscarinic cholinergic signaling in cortical neurons and cells SN56 derived from basal cholinergic neurons [122,123].

Another mechanism that can contribute to synaptic dysfunction in AD is due to downregulation of the 2-amino-3-hydroxy-5-methylisoxazole-4-propionic acid (AMPA) and NMDA receptors. In fact, Aβ-mediated cleavage of AMPA subtype of glutamate receptors has been shown to occur in a caspase-dependent manner both in AD brains and in cultured neurons [124], while recently Aβ has been found to promote the endocytosis of NMDA receptors through a mechanism involving Ca^2+ ^influx and dephosphorylation of the NR2B subunit [125].

Besides its role in synaptic dysfunction and apoptosis, Aβ also contributes to the formation of NFTs of tau, a protein that binds and stabilizes microtubules. Hyperphosphorylation of tau results in the detachments of PHFtau from microtubules and its aggregation as paired helical filaments of the NFTs, thus leading to microtubule destabilization, disruption of the axonal transport and dysfunction of organelles like the Golgi apparatus. Furthermore, as described in paragraph 5.5, both Aβ and PHFtau production are increased by a mechanism involving Wnt signaling and glycogen synthase kinase-3β (GSK-3β) [126,127], whose activity is enhanced in AD brains [128], thus providing a link between the two principal structural and pathological components of AD and decreased trophic support [129].

Based on the evidence supporting the Aβ hypothesis, potential therapeutic strategies have been developed involving immuno-therapy or pharmacological modulation of Aβ production. Both these approaches have also led to some phase I and II clinical trials, whose results indicate a reduction of Aβ levels in the plasma, but not in the cerebrospinal fluid, with γ-secretase inhibitors, and some cases of plaques regression following vaccination [130,131].

## 5. Key players in neuronal apoptosis

Current knowledge of the AD pathology indicate that multiple factors, genetic and environmental, play different roles in determining the conditions that trigger the dysfunction of the cholinergic neurons and the dynamic and coordinated activation of a series of cascades that eventually lead to neuronal death. Before analyzing the distinct events triggering apoptosis in AD, it should be emphasized that most of them include oxidative stress and mitochondrial dysfunction. Thus, these organelles play a key role in the amplification of responses to apoptotic stimuli and, therefore, in cell death decision.

The main groups of molecules involved in the apoptotic cascade include molecules as different as Bcl-2 family members [60], caspases [61,67], adaptor proteins controlling the activation of initiator caspases, such as the Apoptotic Protease Activating Factor-1 (APAF-1), and members of the Tumor Necrosis Factor (TNF) receptor family containing death domains (DD) (p75 and CD95/Fas) [63,132,133], as well transcription factors, proteins involved in the regulation of cell cycle and growth arrest [134-136], heat-shock proteins [137], etc. An important issue is that all these factors are activated through independent signaling pathways whose molecular components are engaged in a complex cross-talk, thus influencing each other in a positive or negative manner.

The Bcl-2 family includes pro-apoptotic (Bax and Bad) and anti-apoptotic (Bcl-2 and Bcl-X_L_) proteins, whose expression and activity is regulated through different signaling pathways including trophic factors deprivation, Aβ toxicity, cytokines and DD receptors activation (Fas and p75) [60,132,138-140]. The central element of the apoptotic cascades is the activation of caspases [61,67,141], a family of cystein proteases (initiator and effector caspases) that can activate DNase and promote the cleavage of enzymes, cytoskeletal proteins (actin and spectrin, fodrin) and ion channels, such as the AMPA receptors [124,140]. Among other proteases that are activated in AD, calpains are activated by increased intracellular Ca^2+ ^levels [142].

All these molecules are involved in two main apoptotic pathways. The intrinsic pathway is centered on mitochondrial dysfunction and is regulated by Bcl-2/Bax proteins [60,139,143]. Traslocation of Bax homodimers to mitochondria leads to release of cytochrome *C *to the cytosol and its binding to APAF-1 with formation of the apoptosome and recruitment of procaspase-9. Oligomerization of APAF-1 promotes the allosteric activation of caspase-9. This initiator caspase can in turn activate the executioner caspases -3 and -7, thus leading to activation of proteolysis and DNA fragmentation. This pathway is activated by most of the known apoptotic triggers in AD, also because is central to cell death caused by oxidative stress.

An extrinsic pathway is, instead, activated by DD receptors [63,132,133]. For instance, interaction of Fas ligand (Fas L) with Fas/CD95 through association with an intracellular adaptor protein Fas-Associated Death Domain (FADD) is followed by activation of the initiator caspase-8 and the effector caspase-3 [141]. The two pathways are not mutually exclusive as, for instance, the mitochondrial intrinsic cascade can also be activated by caspase 8- mediated cleavage of Bid into a truncated tBid, followed by its traslocation to mitochondria where it causes the release of cytochrome *C *[144,145], as well the Apoptosis-Inducing Factor (AIF) which mediates a caspase-independent pathway [146,147].

### 5.1 Does NGF deprivation or proNGF play a role in AD apoptosis?

While NGF-mediated apoptosis during the development is well established [68,69], the role of NGF on survival of cholinergic neurons in the adult is recently gaining stronger experimental support [57,85]. In fact, besides the role of reduced retrograde transport of NGF [36,83] and the new evidence obtained in adult anti-NGF transgenic mice directly linking NGF deprivation to cholinergic deficits [57,85], there might be also a potential role of proNGF in triggering cell death through the p75 receptor [90] whose levels are increased in AD patients along with decreased TrkA expression [95,96].

The aim of this review is the construction of a molecular model of apoptosis in AD. Given the existence of a cross-talk between the apoptotic cascades activated by different apoptotic triggers and that their molecular components are involved in reciprocal modulation by the NGF signaling, we will first review the main molecular mechanisms underlying the control of neuronal survival and death by NGF/proNGF through their interaction with the TrkA and p75 receptors. Co-expression of the two receptors determines the formation of high affinity binding sites that potentiate TrkA signaling under conditions of low NGF concentration, however both receptors can also activate independent signaling cascades leading to survival or apoptosis, depending on cell context [rev. in ref. [148]].

#### TrkA signaling

The role of TrkA signaling in neuronal survival is known to be mediated by receptor dimerization and autophosphorylation on Tyr-490 and Tyr-785. Recruitment to p-Tyr490 of shc (src homologous and collagen-like) and other adaptor proteins is critical to the activation of two independent pathways: ras-Extracellular signal-Regulated Kinases 1/2 (ERK1/2) and Phosphatidylinositol-3 Kinase (PI3K)/Akt (Protein kinase B) pathways.

Ras-ERK1/2 activation leads to ERK1/2 translocation to the nucleus where they phosporylate Elk-1 and regulate the activity of transcription factors and co-activators, such as *c-fos *and CREB Binding Protein (CBP), involved in the control of gene expression. ERK1/2 can also phosphorylate Rsk (Ribosomal S6 Kinase), a cell cycle-regulated kinase that phosphorylates the S6 protein of the 40S ribosomal subunit. Rsk can in turn phosphorylate the IkBα subunit of Nuclear Factor-Kappa B (NF-kB) and CREB, whose activity is known to regulate genes involved in neurite outgrowth, synaptic plasticity and cell cycle.

The PI3K/Akt pathway, instead, regulates neuronal survival by modulating the phosphorylation of members of the bcl-2 family. Akt-mediated phosphorylation of Bad favors its interaction with 14-3-3 protein and prevents it from binding to Bcl-X_L_. As a consequence, Bcl-X_L _is free to bind Bax, thus preventing the formation of Bax homodimers that are pro-apoptotic. In fact, Bax homodimers traslocate to mitochondria and determine the alteration of the membrane potential and release of cytochrome *C *[149]. Akt-mediated survival has been shown to involve also phosphorylation and inhibition of procaspase-9 [150].

Other important substrates of Akt include Forkhead (FKH) transcription factors, GSK-3β and p53 [151-153]. Non-phosphorylated FKHs proteins are localized in the nucleus and activate gene transcription, while phosphorylation by Akt results in their association to 14-3-3 protein and retention in the cytoplasm [151]. FKHs proteins regulate genes controlling cell cycle, cell metabolism, oxidative stress and cell death, such as Fas L, which has been shown to be overexpressed in AD brains [63] and involved in Aβ-induced apoptosis in neuronal cultures [63].

FKHs proteins have also been found to be phosphorylated by a mechanism involving Jun N-terminal Kinase (JNK) and p66Shc during Aβ-induced apoptosis [154], a process that seems to occur also *in vivo*. It has been suggested that relocation of FKHs to the cytosol would result in down-regulation of target genes, such as Mn-Superoxide dismutase (MnSOD), thus leading to increased accumulation of reactive oxigen species (ROS), oxidative stress and cell death. Also GSK-3β, which has been shown to be involved in apoptosis following NGF deprivation and Aβ neurotoxicity [128,129], is inactivated by phosphorylation through the PI3K/Akt signaling [152], as well by Wnt-mediated activation of PKC [155].

A second survival element of the PI3K/Akt pathway is through inhibition of the JNK signaling, as well as the activation of NF-kB. Phosphorylation and degradation of IkB, the inhibitory subunit that sequesters the NF-kB complex in the cytoplasm, releases NF-kB which can traslocate to the nucleus and activate the transcription of genes that promote resistance to apoptosis, such as MnSOD, Bcl-2 and members of the Inhibitor of Apoptosis Proteins (IAPs) family.

Phosphorylation of Tyr785 of TrkA receptors determines, instead, activation of phospholipase C-γ (PLC-γ) and production of two intracellular second messenger: diacylglycerol (DAG), a potent activator of PKC, and Inositol 1,4,5-P_3_(IP3) which stimulates the release of Ca^2+ ^from the endoplasmic reticulum (ER) and the activity of Ca^2+^-dependent proteins (Ca^2+^-calmodulin protein kinase) and phosphatases.

Experimental evidence of cell death following NGF deprivation have been obtained *in vitro*, mainly in PC12 cells and sympathetic neurons, where apoptosis following growth factor deprivation occurs mainly through an intrinsic pathway activated upon a failure of the TrkA/PI3K/Akt signaling, followed by translocation of Bax to mitochondria and release of cytochrome *C *[138,139,156]. Decreased TrkA/PI3K/Akt signaling would also result in the activation of JNK and induction of p53 [153], as well FKH-mediated transcription [151]. Although NGF deprivation has been found to induce Fas and Fas L expression, activation of an extrinsic pathway may contribute, but does not seem to be required for apoptosis after NGF deprivation [139].

#### p75 signaling

The p75 receptor belongs to the TNF receptor family because of the presence of a DD in the cytoplasmic region. This property confers to this receptor the ability to control cell survival/apoptosis depending on cell context, mainly the presence/absence of TrkA receptors [rev. in ref. [148]].

Increasing evidence has been accumulated so far about the role of p75 in neuronal death both *in vitro *and *in vivo *[93,153,157]. For instance, p75 has been shown to mediate apoptosis of developing sympathetic neurons [153], cultured cortical and hippocampal neurons [157], as well in mature oligodendrocytes [158]. Moreover, overexpression of p75 contributes to neuronal death of cholinergic neurons [93]. However, the precise molecular mechanisms of the p75-mediated cell death are still controversial. In fact, regardless of the presence of a DD, the apoptotic cascade is quite different from that induced by other TNF receptors family members. Signaling through p75 does not appear to activate caspase-8, the end point of a typical extrinsic pathway induced by Fas and other related members of the TNF receptors.

Several adaptor proteins have been found to interact with the DD of p75, including: NRIF1/2 (Neurotrophin-Receptor Interacting Factor 1 and 2) and NADE (Neurotrophin-Associated cell Death Executor) both involved in apoptosis; SC-1 (Schwann cell-1) and NRAGE (Neurotrophin Receptor-interacting MAGE homologue) that are associated to growth arrest and cell cycle events; RIP2 (Receptor-Interacting Protein 2), which possesses a CARD (Caspase Recruitment Domain) and TRAF-4/6 (TNF Receptor Associated Factor-4 and -6), both linked to survival responses [159-161].

The p75-mediated apoptosis have been found to occur mainly through activation of the sphingomyelinase/ceramide and JNK pathways [162]. The precise mechanism underlying sphingomyelinase activation by p75 is still unclear, but in oligodendrocytes there is a strict correlation between p75-mediated generation of ceramide and rac/JNK activation [158,163]. It is intriguing that ceramide has been shown to directly inhibit ERK and PI3K/Akt signaling [164,165] thus evoking the same apoptotic cascade induced by NGF deprivation. This inhibition is part of the well known reciprocal regulation between the two NGF receptors, as ceramide signaling is also inhibited by PI3K/Akt. In cortical neurons ceramide induces activation of the mitochondrial apoptotic cascade and is associated to dephosphorylation of Akt, BAD, FKH and GSK-3β [165].

The mitochondrial intrinsic pathway following p75 signaling is also mediated by the JNK pathway through stabilization of p53 [153] which targets several genes, including Bax. Thus, p53 is a common component of the pathways activated by the two NGF receptors, both TrkA following NGF withdrawal and p75 signaling. The mitochondrial cascade leading to activation of the initiator caspase -9, and then caspases -3 and -7, has been found to be mediated by a JNK-dependent pathway following interaction of p75 with NRAGE [159]. JNK/p53 also triggers the induction of Fas L which promotes apoptosis through binding to Fas receptor [132]. Fas L and Fas receptor expression are elevated both in AD and in cultured neurons treated with Aβ [63].

Another target of p75 signaling is the activation of NF-kB [166]. Recently, *in vitro *studies have shown that NF-kB activation can be mediated by the interaction of p75 with RIP2 and TRAF-6 [160,161]. TRAF-6 activates the protein kinase NFkB-Interacting Kinase (NIK), which phosphorylates the Inhibitor of IkB Kinase (IKK), thus leading to phosphorylation and degradation of IkB and translocation of NF-kB to the nucleus.

Induction of NF-kB by p75-mediated signaling has been observed in many cell types, under different conditions [166,167] and mostly linked to a survival function. In general, NF-kB is regarded as an anti-apoptotic transcription factor and its activation is believed to be fundamental in neuroprotection and repair following brain injury and upregulation of p75 expression. Thus, it is reasonable to believe that activation of this transcription factor is one of the mechanisms through which cells are able to control between life and death decisions.

Finally, p75^NTR ^receptor has been found to directly interact with RhoA, a member of the Ras-GTP binding proteins and a regulator of actin assembly in many cell types, suggesting a role for this receptor in the neurite outgrowth and reorganization of the actin cytoscheleton, a function that is negatively modulated by neurotrophin binding [168].

### 5.2 Aβ toxicity and oxidative stress

Oxidative stress and mitochondrial dysfunction are common players in the apoptotic cascades leading to neuronal death in AD and are induced by all the known triggers, including growth factor deprivation and Aβ toxicity both *in vitro *and *in vivo *[37,169]. Aβ can induce apoptosis directly by increasing generation of ROS and mitochondrial dysfunction. ROS accumulation determines oxidation of proteins, peroxidation of membrane lipids and DNA damage. Oxidative stress causes then a dysfunction of Na^+^/K^+^- and Ca^2+^-ATPases, alteration of glucose and glutamate transport, excessive Ca^2+ ^influx, ATP depletion, membrane depolarization and translocation of cytochrome *C *to the cytoplasm [rev. in ref [42]].

It should be briefly mentioned that further contribution to oxidative stress is also given by Fe^2+^, Cu^2+ ^and Zn^2+ ^ions [42], whose levels are increased in the senile plaques of AD patients. Several *in vitro *and *in vivo *studies have demonstrated that these ions are implicated in Aβ deposition [170].

Besides the evidence demonstrating the involvement of Aβ in the impairment of synaptic activity, Aβ toxicity has been found to be a central element of the apoptotic neuronal death in AD, as extensively demonstrated both *in vivo *[37,48,62,63] and *in vitro *by using either primary cultured neurons or PC12 cells [59,66,171]. Aβ-induced apoptosis of cortical neurons is characterized by internucleosomal fragmentation after only a 6 hr-exposure to Aβ and is greatly increased by expression of p75 [172-174], thus linking Aβ toxicity to the aberrant expression of p75 and TrkA in AD [95,96].

Aβ-induced apoptosis is mediated by activation of the Stress-activated protein kinases (SAPK) p38 Mitogen Activated Protein Kinase (p38MAPK) and JNK, followed by activation of both NF-kB and p53 [171,174,175]. Active forms of JNK and p38 kinase have been found in hippocampal and cortical neurons of AD brains, suggesting their potential implication in hyperphosphorylation of tau and NFT formation [176].

Recently, p75-mediated cell death in Aβ neurotoxicity has also been shown to occur through interaction of p75 with a newly identified P75-Like Apoptosis-Inducing Death Domain (PLAIDD) [177]. Moreover, a new protein has been identified, Aβ-Binding Alcohol Dehydrogenase (ABAD), that is highly expressed in the brain and has been clearly shown to link Aβ-toxicity to the mitochondrial oxidative stress [178].

In hippocampal neurons, sympathetic neurons and PC12 cells, Aβ-toxicity has been found to be mediated by activation caspases-9 and -3, as well by caspase-2 [171,179].

In AD, caspases -9, -3, -6 and -8 colocalize with NFT, suggesting a direct role of caspases in cleavage of tau and formation of NFT [61,67,180]. Caspase-8 in the brain of AD patients has been also shown to be induced through a mechanism mediated by the TNF-Related Apoptosis-Inducing Ligand (TRAIL) in response to Aβ toxicity. Evidence from *in vitro *studies also indicates that TRAIL might provide a substantial contribution to the progression of the neurodegeneration during chronic inflammatory responses [181]. Other caspases that are found overexpressed in postmortem brains from AD patients include caspases-2, -5 and -7 [62].

However, besides the strong presence of the many activated caspases, also increased NF-kB activity has been observed in neurons and glia of brain sections from AD patients, as well in cultured primary neurons exposed to Aβ [182]. Thus, it is quite clear that also *in vivo *apoptosis involves activation of several apoptotic cascades, as well as signaling pathways that lead to trascription of neuroprotective genes in an attempt to rescue neurons from cell death.

### 5.3 Excitotoxicity: role of Ca^2+ ^and calpains in cell death and NFT formation

As previously mentioned, excitotoxicity plays a role in many acute and chronic degenerative diseases and exacerbates oxidative stress through a mechanism leading to disruption of Ca^2+ ^homeostasis and increase of ROS [183].

Ca^2+ ^is a critical regulator of synaptic plasticity because of its role in LTP and LTD, postranslational modification of proteins involved in neurite elongation and synaptic activity (actin-binding proteins and ion channels), activation of kinases, such as Protein Kinase A (PKA), Calcium-Calmodulin Kinase II (CaMKII), and ras-Mitogen Activated Kinase (MAPK), as well as the activity of transcription factors, like Elk-1, CREB and Activator Protein-1 (AP-1) [184]. Moreover, cytosolic free Ca^2+ ^mobilized from the intracellular stores of the ER has been shown to be also a second messenger for NGF-TrkA signaling linked to the activation of PLC-γ [185]. However, persistent alterations of Ca^2+ ^homeostasis following excitotoxicity and oxidative stress can contribute to neuronal apoptosis in AD [142,186]. One target for increased intracellular Ca^2+ ^levels are calpains [142], cystein proteases involved in the regulation of cytoskeletal remodeling. Calpains were found to participate to apoptosis through cleavage of Bax [186], followed by release of cytochrome *C *and activation of caspase-3, as well as by direct activation of caspases-7 and -12.

Calpain activation has been detected in the brains of AD patients in proximity of NFT [187] and suggested to play a role in the regulation of tau phosphorylation and NFT formation by a mechanism involving the cleavage of p35 to p25 [188] and activation of cdk5 [188,189]. Cdk5, a member of the cyclin-dependent kinase family not linked to cell cycle, is highly expressed in neurons and, together with its regulatory protein p35, is required for neurite outgrowth. In AD, accumulation of p25 correlated with enhanced cdk5 kinase activity, while expression of p25/cdk5 in cortical neurons was found to cause increased tau phosphorylation, disruption of cytoskeleton and apoptosis [189]. Inhibition of cdk5 or calpain activity reduced cell death in Aβ-treated cortical neurons, suggesting that the p25/cdk5 complex mediates also Aβ-neurotoxicity [188].

On the other hand, there is increasing evidence showing that NFT formation and apoptosis might also involve an impairment of the ubiquitin-proteasome (UB-proteasome) system, whose activity is required for degradation of short-lived and damaged proteins, such as misfolded or modified proteins in AD. *In vitro *studies have shown that failure of the proteasome system is an early event in several apoptotic conditions including NGF deprivation of sympathetic neurons [190] and exposure of cerebellar granule cells to reduced K^+ ^levels [191]. The latter has been found to be associated with caspase-3 activation and accumulation of ubiquitinated proteins in the cytosol [191].

There is evidence for impaiment of the UB-proteasome system also in AD. Among the others, a mutant form of ubiquitin (Ub^+1^) has been found in the brain of AD patients, that would result in dominant inhibition of the UB-proteasome system [192] and might play a critical role in accumulation of misfolded tau proteins forming NFT.

### 5.4 Vascular factors and brain metabolism

A contribution to increased Aβ production and deposition was found in individuals carrying the ε4 allele of the Apolipoprotein E (APOE) gene. APOE is a plasma lipoprotein that functions as the major cholesterol carrier to the brain, thus linking hypercholesterolaemia and alteration of brain cholesterol homeostasis to the risk of developing AD. This correlation has been suggested also by several epidemiological studies, as well by the evidence that statins, which reduce cholesterol levels, also decrease the risk of AD [rev. in refs [193,194]].

Atherosclerosis is also a risk factor for AD, as reduced cerebral blood flow can cause reduced glucose utilization in AD patients [195]. Other vascular factors in AD include diabetes and alteration of serum levels of insulin and Insulin Growth Factor-1 (IGF-1), two important regulators of brain energy balance. In fact, IGF-1 has been found to be implicated in modulation of synaptic plasticity [196,197], regulation of Aβ levels [198] and neuroprotection against Aβ toxicity [199] by a mechanism involving Akt and GSK-3β [200,201].

Insulin/IGF-1 serum levels and signaling are impaired in AD patients suggesting a link also between AD pathology and defective signaling by these two important regulators of the brain energy balance. Thus, it is conceivable that a decline in metabolic and hormonal function in aging, together with accumulation of oxidative stress, can influence neuronal vulnerability to other concomitant insults and result in neuronal degeneration through activation of apoptotic cascades.

### 5.5 IGF-1, Wnt signaling and GSK-3β

The relevance of IGF-1 in neuroprotection developes around the activity GSK-3β, which is a central element of the IGF-1/Wnt signaling, and also a target of the PI3K/Akt pathway.

AD is characterized by increased levels of GSK-3β which, in its active (dephosphorylated) state, is a key regulator of NFT formation [126]. GSK-3β is phosphorylated (and inactivated) through a PI3K/Akt signaling pathway induced by IGF-1, NGF and any growth factor whose activity is mediated by receptors with intrinsic tyrosine kinase activity [201], including also Fibroblast Growth Factor-2 (FGF-2). Down-regulation of PI3K/Akt results in active GSK-3β and renders neurons more vulnerable to Aβ/JNK/p53 cascade. In fact, GSK-3β is activated, along with caspases, during NGF deprivation-induced cell death of PC12 cells [129], Aβ- toxicity and oxidative stress [152] by a mechanism mediated by reduced PI3K/Akt signaling and JNK/p53 activation, thus providing a link between NGF deprivation and the two pathological components of AD.

Among other evidence indicating a role in neuroprotection, IGF-1, NGF and FGF-2 have been shown to protect hippocampal neurons against Aβ toxicity [199,201-203].

GSK-3β is also inactivated by the mitogenic IGF-1/Wnt pathway. GSK-3β inactivation results in: *i*) decreased tau phosphorylation and *ii*) accumulation of β-catenin followed by translocation to the nucleus and regulation of cyclin D1 expression and c-myc [204]. The Wnt signaling can be inhibited by p53 leading to active GSK-3β which determines phosphorylation of β-catenin for degradation by the ubiquitin-proteosome system, increased Aβ production and increased tau phosphorylation. Thus, GSK-3β provides also a link between decreased IGF-1-mediated trophic support and Aβ toxicity/tau phosphorylation/cell cycle events [152].

### 5.6 Apoptosis and cell cycle events

An intriguing issue about the apoptotic processes underlying neurodegenerative diseases is raised by the question that neuronal death is accompanied by cell cycle events, suggesting that neurons attempt to divide before dying. Moreover, there is increasing evidence that neuronal apoptosis can arise from a failure of degenerating neurons to re-entry the cell cycle. Mature neurons exit the cell cycle and remain quiescent in the G0 phase, however, active cell cycle proteins are present in neurons of AD brain, including cyclins, cell cycle kinases, as well as their activators and inhibitors [134-136,205,206]. Cell cycle proteins and complexes found in AD brains include cyclinD, cdk4 and Ki67, as well the presence of cyclinE/cdk2 which controls G1/S phase transition, indicating an alteration of cell cycle events in AD neurons.

An increase in cyclin D1 expression has been demonstrated also in Aβ-induced apoptosis of differentiated neurons [206]. In addition, several *in vitro *studies have shown that apoptosis induced by NGF deprivation occurs together with the expression of cell cycle proteins cdk4 and cdk6 and their cognate cyclins, and that cdk inhibitors p16^Ink4^, p21^Cip1 ^and p27^Kip1^, as well the DN-cdk4/6, promoted survival of differentiated PC12 cells and sympathetic neurons [207,208]. Besides supporting survival, p16^Ink4 ^and p21^Cip1 ^have been shown to reconstitute NGF-dependent neurite outgrowth in PC12 cells [209].

Similarly, elevated expression of the cdk inhibitor p16^Ink4 ^and other members of the INK4-family (p15^Ink4b^, p18^Ink4c ^and p19^Ink4d^), which bind complexes of cdk4/6-cyclinD, were found also in AD, mainly in neurons with NFT and neuritic plaques [135]. Several other studies have demonstrated increased expression of cell cycle proteins, including Proliferating Cell Nuclear Antigen (PCNA), cyclins B, C and D in the brain of AD patients, both in neurons and astrocytes. Moreover, cell cycle events appear to occur at early stages of the disease (in MCI) and precede neuronal cell death [136,210,211].

Many factors may induce neurons to attempt a cell cycle re-entry. One is the overexpression of growth factors with mitogenic/differentiation activity, such as NGF, IGF-1/2 and Epidermal Growth factor (EGF), as well Tumor Growth Factor-α 1 (TGFα 1) and FGF-2 secreted by reactive astrocytes and microglia in response to brain damage. Most of these growth factors signal through the ras-MAPK pathway [212] that is involved in the regulation of G0/G1 transition. At an early stage of AD, elevated expression of p21ras co-localizes with p16^Ink4 ^in pyramidal neurons [213]. ERK activation is also linked to c-myc phosphorylation in AD.

Cell cycle re-entry may occur also through mechanisms mediated by GSK-3β following phosphorylation (and inactivation) by either a PI3K/Akt signaling or the mitogenic Wnt pathway that is a central element of IGF-1 signaling. For instance, the inactivation of GSK-3β results in the upregulation of cyclin D1 expression in carcinoma cells [204].

Furthermore, cell cycle re-entry might also be triggered by oxidative stress through activation of the stress-activated protein kinases JNK-SAPK and p38-SAPK2, which control stress responses in AD [176], suggesting a possible link between the occurrence of cell cycle reentry and increased oxidative stress and neuronal vulnerability in aging. Accordingly, neurons in AD brains are characterized by extensive DNA damage and increased p53 expression. However, in PC12 cells and cortical neurons, exposure to Aβ induces upregulation of cdk4/6, phosphorylation of the retinoblastoma protein pRb/107, release of Rb from the transcription factor complex E2F-DP and activation of E2F-mediated trascription of genes required for S phase transition, but these events occur in a p53-independent manner [214].

Cell cycle components have been proved to be expressed also in FAD mutants of APP, where cell cycle entry can be mediated by the APP-binding protein 1 (APP-BP1) [215] or p21-Activated Kinase3 (PAK3) [216]. Recently, a reduced PAK pathway was associated to cognitive deficits in AD [217].

### 5.7 Inflammatory processes

Finally, another relevant factor in the pathogenesis of AD is given by neuroinflammatory processes, a common response to brain damage mediated by the proinflammatory cytokines Interleukin(IL)-1α (IL-1α), IL-1β, TNFα and TGF-β 1, chemokines and the prostaglandin E2 (PGE2) released by reactive astrocytes [218].

Many of these inflammatory mediators have been shown to mediate protection by inducing the synthesis of growth factor. For instance, NGF while under normal conditions is localized in neurons, during inflammation is synthesized also by microglia and reactive astrocytes, where it is upregulated by cytokines (IL-1α, IL-1β and IL-6) and other inflammatory mediators, such as Interferon-γ (IFN-γ) and Lipopolysaccharide (LPS) [reviewed in ref. [107]]. This is a common response to CNS injury and it is linked to the role of NGF in neuroprotection and repair. NGF-mediated activation of both TrkA and p75, the latter being increased following brain damage, may promote neuronal survival through activation of NF-kB [182], which can also be directly induced by most of the cytokines.

Microglial activation have also a phagocytosis function for removal of apoptotic cells, thus limiting the spread of toxins to the adjacent tissues. Neverthless, it is believed that the inflammatory processes contribute to exacerbation of the AD pathology and rapid progression of the disease. The activation of microglia causes chronic inflammation by inducing an autotoxic loop due to secretion of proinflammatory cytokines (TNFα and IL-1β), acute-phase proteins and overproduction of free radicals (nitric oxide and superoxide) [218,219]. An amplification of the inflammatory processes is also due to activation of the complement system that has been shown to be also directly induced by Aβ [220].

The role of inflammatory processes in AD progression may explain the protection by non- steroid anti-inflammatory drugs observed in epidemiological studies [221], due to both cyclooxygenase-1 and -2 (COX1-2) inhibition of PGE2 synthesis and modulation of γ-secretase [222]. A different mechanism of protection may instead be mediated by the naturally occuring anti-inflammatory cytokines, such as IL-10 which has been shown to prevent glutamate toxicity through inhibition of caspase-3 [223].

## 6. Toward a blueprint of neuronal apoptosis

It is clear from the experimental evidence summarized in the two previous paragraphs that neuronal apoptosis in AD is quite a complex process, thus posing many problems to the current efforts of acquiring a comprehensive understanding of the phenomenon and ordering of the sequential events.

Recently, attempts to obtain this information have involved different approaches, including microarray analysis of expression profiles [64,65]. One of these studies revealed that Aβ-induced apoptosis of cultured cortical neurons involves the differential expression of 956 genes, and 70 of them were common to those whose expression was altered during apoptosis of cerebellar granule cells by serum and potassium deprivation as well [64]. These genes included neurotransmitter receptors, neuropeptides, ion channels, growth factors, transcription factors, enzymes, structural proteins, and so on. These data strongly suggest that neuronal apoptosis displays several regulatory mechanisms that are common to other apoptotic processes triggered by various insults and in a cell type-independent manner.

Neverthless, gene expression analysis can only provide a partial picture of the whole process. The notion is well accepted that apoptosis is an active process requiring *de novo *protein synthesis [224]. However, many events are based on translational or posttranslational modifications, such as allosteric modifications, phosphorylations, compartimentalization, as well as cellular intermediates that belong to different classes of molecules, such as Ca^2+^, ceramide, etc.

As mentioned in the first paragraph of this review, a modular systems biology approach has been shown to be a useful strategy for organizing information and providing molecular models of complex bioprocesses. A similar instrumental benefit might be obtained for apoptosis in chronic neurodegenerative diseases.

**Figure 2:**
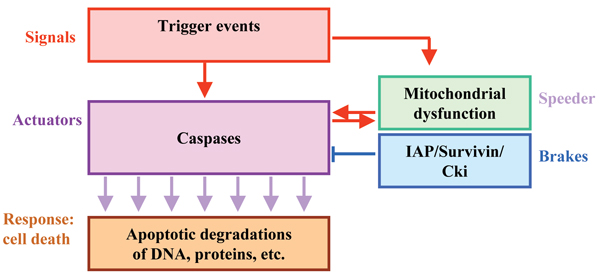
The basic blueprint of neuronal apoptosis showing the different modules and how they are interconnected by positive and negative feed-backs.

Given the complexity of the phenomenon in AD, a large body of data needs to be organized and structured first in a low-level model (a blueprint) and further in a more detailed molecular model. A recent mathematical model of CD95-induced apoptosis [28] is used in this review as backbone to start to organize the data gathered on neuronal apoptosis. The proposed basic blueprint for neuronal apoptosis shown in Figure 2 considers a trigger submodule (the DISC-system in the Bentele *et al*. paper), an actuator (the caspase submodule), a feed-forward module (mitochondrial dysfunction), a brake (IAP/Survivin/Cki) and the many degradative responses of apoptosis (degradation of proteins, poly(ADP-ribose)polymerase (PARP), DNA and so on).

As shown in Figure 2, the structure of the basic blueprint indicates quite clearly that the activation of caspases is due to one specific event, although it depends on various triggers. The mitochondria submodule, that has a feed-forward effect on caspases, may be further stimulated by an autocatalytic process, while a negative feed-back on caspases depends on the IAP/Survivin/Cki module. The fully fledged caspases induce apoptotic death by degrading the various cellular components.

The structure of the blueprint makes it possible to analyze the condition in which the caspases activation is not fully expressed and investigate the role that both the IAP/Survivin/Cki module and the anti-apoptotic molecules acting on mitochondria may have in protecting neurons from death.

Let us now go through the various submodules of the blueprint and analyze how the findings summarized in paragraphs 4 and 5 fit with the picture.

### 6.1. The triggers

**Figure 3:**
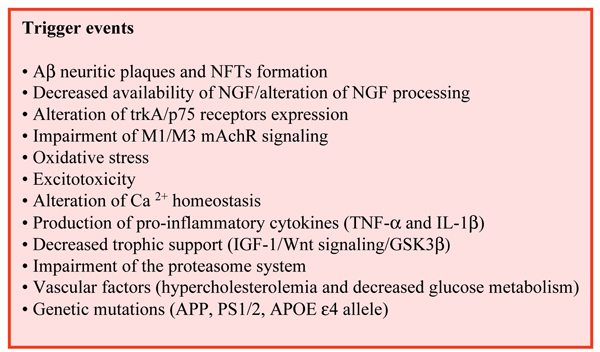
A list of the many genetic and environmental events that contribute to the activation of apoptotic cascades in AD neurodegeneration.

It is quite clear from Figure 3 that the events able to trigger neuronal programmed cell death are numerous and quite diverse, and possibly in cross-talk.

Because of its pivotal role for maintenance of the cholinergic phenotype, one of the key triggers might be the alteration of NGF availability and/or transport, and failure of the TrkA-mediated signaling (via ras-ERK1/2 and PI3K pathways), as described in paragraphs 4.1 and 5.1. On the other hand, increased pro-NGF levels in AD [90] might also play an important role in triggering neuronal apoptosis through p75 receptor [91], whose upregulation is common to a variety of neuronal injuries. Indeed, although the precise p75 signaling is not completely understood, p75 can induce apoptosis through a neurotrophin-dependent activation of the sphingomyelinase/ceramide and JNK/p53 pathways followed by induction of Bax and Fas L, thus involving the activation of the mitochondrial intrinsic cascade and, perhaps, also an extrinsic apoptotic pathway, given that Fas and FasL have been found in senile plaques in AD and suggested to participate Aβ-induced neuronal death [63].

Aβ is a another key trigger of neuronal apoptosis in AD and its neurotoxicity is enhanced by expression of the p75 receptor. Besides the involvement of the ceramide/JNK pathway, Aβ toxicity leads to accumulation of ROS, thus further increasing a condition of oxidative stress that is peculiar to the brain in aging and represents the third main element strongly implicated in neuronal apoptosis in AD, through activation of the mitochondrial intrinsic cascade. Moreover, ROS-mediated activation of caspase-3, can further stimulate sphingomyelinase and ceramide production, thus generating a feed-forward loop. An extrinsic pathway is also activated by Aβ through the induction of TRAIL and activation of caspase-8.

As mentioned, an important role in neuroprotection is played by other growth factors, such as FGF-2 and IGF-1, meaning that availability of these growth factors can influence the balance of pro- and anti-apoptotic mediators (Bcl-2/Bax, caspases, NF-kB, etc.) and push toward the rescue of cells rather than their death. The relevance of trophic support, mainly NGF and IGF-1, and other growth factor signaling through trk receptors and activation of PI3K/Akt and PKC, is also linked to the key role played by GSK-3β in the regulation of both Aβ accumulation and NFT formation, as well to cell cycle events.

Another important element is the alteration of Ca^2+ ^homeostasis that can be caused by both oxidative stress and excitotoxicity, a common pathway in many neuropathological conditions [35]. Besides the role in cell death, Ca^2+ ^gives a further contribution to the formation of NFT by activating calpains and p25-cdk5 complexes which increase tau phosphorylation [188,189].

Moreover, among the other mechanisms triggering cell death and described in paragraph 4, we must acknowledge also the genetic mutations (APP, PS1/2, APOE) that cause an alteration of APP processing and accumulation of neurotoxic Aβ, although the role of the genetic factors is limited to the familial forms of AD.

### 6.2. The caspases

This module is characterized by one initiator caspase (caspase-8 or caspase-9) activated by the trigger events and several executioner caspases (caspases-3, -6, -7). Caspases are synthesized as inactive pro-caspases that are cleaved at the internal proteolytic site to yield the active caspase. The initiator caspases, once activated, will activate the downstream effector caspases, which in turn will cleave their substrates, such as PARP.

In the extrinsic pathway this module is initiated by the association of the DD receptor to its ligand and recruitment of an adaptor molecule and procaspase-8 to form the DISC (Death Inducing Signaling Complex). Procaspase-8 is then cleaved to active caspase-8 which in turn activates the effector caspase-3 [141]. The extrinsic pathway mediated by DD receptors in AD is believed to be prevalently linked to Fas-FasL, TRAIL and cytokines activity during inflammation.

The intrinsic or "mitochondrial" pathway, instead, includes the activation of the initiator caspase-9 mediated by the heptameric apoptosome complex. Formation of apoptosome is initiated by the interaction of the cytoplasmic APAF-1 with cytochrome *C *derived by the dysfunction of mitochondria. Once recruited to the apoptosome, procaspase-9 acquires autocatalytic activity and then activates also the executioner caspases-3 and -7. Caspase-3 activates, by proteolytic cleavage, both the executioner caspase-6 and caspase-2 which, in turn, activates a feed-forward loop by stimulating mitochondria to release more cytochrome *C *and activate more caspase-9.

**Figure 4:**
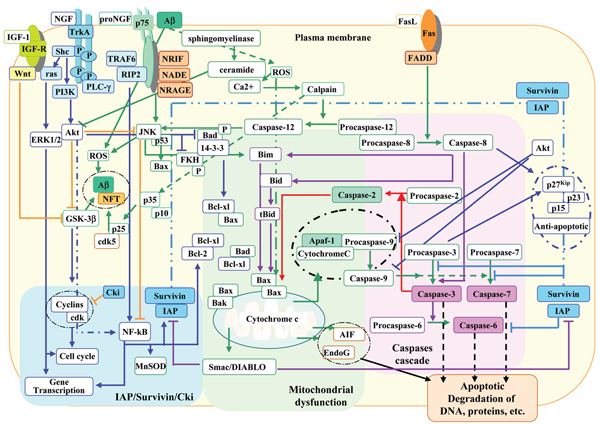
A molecular model of neuronal apoptosis organized in submodules. The central part of the model (green panel) represents the mitochondria submodule and all the molecules that regulate its function as "sensor" of the cellular stress responses (yellow panel). Dark blue and green arrows indicate survival and apoptotic pathways, respectively; orange arrows indicate inhibitions. Red arrows indicate the positive feed-back, while in purple and light blue are indicated the cross-talk between extrinsic/intrinsic apoptotic cascades and the survival regulatory control by IAPs, respectively. More information is the text.

At this point we moved to collect the available information in a molecular model (Figure 4) structured in modules according to Figure 2, although we do not claim it to be exhaustive and definitive, but a useful tool for a systems biology approach. When considering caspases, Figure 4 shows that the overall dynamics of this submodule sees distinct triggers that independently activate pathways of proteolysis that converge on the three executioner caspases, whose activity is strongly controlled by a feed-forward loop acting on mitochondria. This process is in part sustained by autocatalytic events, such as the activation of caspase-2 by caspase-3 involved in the feed-forward loop, as well the activation of caspase-6 by caspase-3 that allows an amplification of the final proteolytic events controlling protein degradation and DNA fragmentation.

In addition, we can observe that in AD the majority of the cell death signals converge on the mitochondrial intrinsic pathway and that the two main caspase cascades are not independent on each other. A cross-talk between the two pathways is mediated by caspase-8, cleavage of Bid and traslocation of tBid to mitochondria where, directly or indirectly, it causes the release of cytochrome *C*. Also this cross-talk between the DD receptor signal and mitochondria leads to an amplification of caspases activation.

### 6.3. The speeder: the mitochondrial submodule

The speeder submodule involves processes that integrate the initial apoptotic signaling which otherwise could proceed to either cell death or recovery from stress events. The main actors are mitochondria which play a crucial role in caspase-dependent and independent apoptotic signaling through the interaction with both pro-apoptotic (Bax, Bak, Bad, Bid, Bik and Bim) and anti-apoptotic proteins (Bcl-2, Bcl-X_L _and Bcl-w) [149]. Following trigger events, their activation and association in different homo- and hetero-dimers function as a "sensor" of the level of cellular stress and damage. Proapoptotic proteins induce cell death by regulating the formation of transition pore (MTP) in the outer mitochondrial membrane. Mitochondrial membrane permeability (MMP) determines the release of cytochrome *C*, Smac/DIABLO, AIF and endonuclease G (Endo G). Traslocation of cytochrome *C *to the cytoplasm is crucial to the activation of the initiator caspase-9 and the executioner caspases-3 and -7, followed by cleavage of PARP and activation of Caspase-activated DNase (CAD) and DNA fragmentation. AIF is, instead, the final effector of a caspase-independent pathway that causes extensive DNA fragmentation, while Smac/DIABLO participate in the final steps of both the extrinsic and intrinsic pathway by inhibiting the IAP proteins. ROS-mediated activation of the intrinsic apoptotic pathway involves the release of several mitochondrial proteins, such as cytochrome *C*, AIF and Smac/DIABLO.

MMP is determined by Bax and Bak homodimers which are formed upon allosteric conformational changes and dimerization in response to trigger signals. Bax and Bak activation is mediated by Bad, Bik, Bid, and Bim and is due to either the preferential interaction with Bcl-2 and Bcl-X_L_, or their release from these anti-apoptotic proteins, thus shifting the balance of the different heterodimers toward the formation of the proapoptotic Bax and Bak homodimers. In sympathetic neurons, following growth factor deprivation and failure of PI3K/Akt signaling, Bad is activated by dephosphorylation, dissociation from the molecular chaperone 14-3-3 and association with Bcl-X_L_. In viable cells, Bcl-X_L _binds Bax and Bak, thus keeping them in an inactive form. Bid and Bim can be also activated by a direct cleavage by caspase-8, as well as by the lysosomal protease cathepsin, in response to ceramide, ROS and JNK.

The model presented in Figure 4 indicates that mitochondria play a significant role in the amplification of the apoptotic processes initiated by growth factor deprivation, DD receptors activation (p75 and Fas), increased levels of intracellular Ca^2+ ^linked to excitotoxicity and ROS accumulation in response to the many brain insults.

### 6.4. The brake: the IAP/Survivin/Cki submodule

In a scenario that mainly sees the propagation of various apoptotic stimuli that may compromise mitochondrial function and converge into the activation of a series of caspases and other effectors of cell death, a brake submodule is given by the IAP proteins (Figure 4). The IAP family of proteins include XIAP (X-linked IAP), c-IAP-1/2 and Survivin which are potent regulators of caspase activity. They function by inhibiting both the activation of pro-caspases and the enzymatic activity of active caspases, such as caspases-3, -7 and -9.

The pro-survival activity of IAPs, however, can in turn be inhibited by Smac/DIABLO that, once released from mitochondria, can bind IAP and promote activation of caspase-3. This modulation represents one of the many examples of the fine regulatory control of apoptosis. An interesting connection between cell cycle and apoptosis is given by the antiapoptotic activity of the Cki p27^Kip1 ^and p21^Cip1^. When these proteins are phosphorylated by Akt, they are translocated to the cytoplasm where they may have antiapoptotic activity [225-227]. More specifically, p27^Kip1 ^has been shown to be cleaved by caspases in the p-23 and p-15 N-terminal peptides that have a strong antiapoptotic activity [226].

Although, as shown above, there is enough information to sketch the lines of structure of the various submodules of the apoptotic process in general, investigations are needed to identify the protein networks actually present in neuronal cells in various neurodegenerative disorders in order to make faithful mathematical models able to yield accurate predictions.

The application of the roadmap of modular systems biology to investigate neuronal apoptosis is expected to improve pathways and network identification with high efficiency.

## 7. Neuroprotection and control of apoptosis

Current therapies for Alzheimer's disease as well as for other neurodegenerative disorders are solely symptomatic [228]. The efforts of the pharmaceutical industry are now focusing on several steps of the hypothesized pathogenic pathways: for instance, prevention of production of neurodegenerative factors (plaques and NFT), targeting mediators of deleterious signaling cascades and trophic support. How does this approach fit with the blueprint of neuronal apoptosis discussed so far?

The concept of neuroprotection implies the reversibility or at least the significant slow down of the apoptotic process. It is quite clear that apoptosis cannot be reversed when a fully fledged activation of executioner caspases has been achieved. It is therefore reasonable to suppose that the passage from cells under apoptotic stress to dying cells requires the full activity of the positive feed-back loop based on mitochondria. What is the role of caspase-2 in this process? Which are the manipulations more likely to damp down the apoptotic stress? What are the markers of mild to severe apoptotic stress? How do the cells of a population/tissue respond to a specific apoptotic trigger? How will the overall activity of the cell, such as metabolic remodeling due to general environmental stress response in chromatin remodeling and ensuing changes in gene expression [229] affect neuronal apoptosis?

The molecular map drawn in Figure 4 attempts to compile the essential molecular pathways that chatacterize neuronal apoptosis. As said before, it does not pretend to be exhaustive, but only opens the way to a systems biology approach of neuronal apoptosis. First of all it presents a trigger module that includes the many genetic and environmental factors listed in Figure 3. Each triggering insult can activate pathways that independently lead to specific modifications/alterations of neuronal homeostasis and eventually initiate a cell death program.

The process is complicated by the existence of an intense cross-talk between the various molecular intermediates that directly or indirectly influence each other, as well as many other parameters controlling cellular homeostasis. The result is the sequential intervention of a large number of metabolic and signaling cascades that may also act synergistically.

Another important element is the redundancy of signalings leading to a specific effect. For instance, NFT formation is induced through activation (dephosphorylation) of GSK-3β due to downregulation of PI3K/Akt, as well as by activation of the p25-cdk5 complex following Ca^2+^-dependent activation of calpains and cleavage of p35, and both pathways can become synergistically activated upon trophic factors deprivation and ROS accumulation, suggesting that cells use more than one route to respond to stimuli.

A further level of amplification is present in the caspase submodule. As shown in Figure 4, caspases are activated both by the many stimuli that trigger the mitochondrial intrinsic cascade (Bax, citochrome C/Apaf-1, caspase-9 and caspases-3/-7), and by the extrinsic pathways (DD receptors, caspase-8, caspase-3). Besides these cascades (green arrows), the caspase submodule is based on three mechanisms: *i*) autocatalytic events leading to the activation of many proteolytic enzymes that, once activated, greatly accelerate the degradation processes; *ii*) a feed-forward loop based on mitochondria and involving caspases and Bcl-2 family members (red arrows), and *iii*) a cross-talk between the extrinsic and intrinsic cascades (purple arrows).

**Figure 5:**
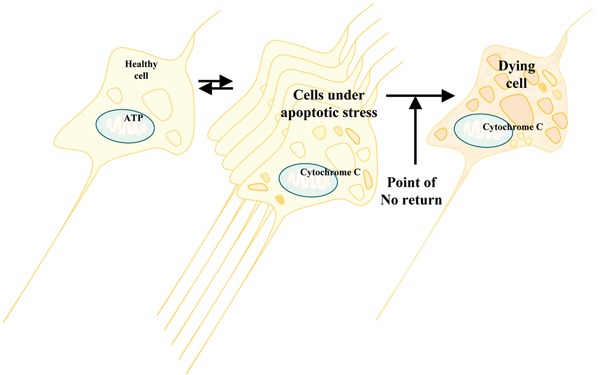
A drawing exemplifying the active "stand-by" status of cells exposed to the various stressful events involved in AD apoptosis. Neuronal death occurs when the cell(s) reaches a point of "No-return", where an overwhelming mitochondria pressure is no longer compensated by the many protective pathways that the cell(s) has adopted.

This evidence indicates that mitochondria, besides playing an essential role in providing energy for all the cellular activities, also represent the central element of the apoptotic machinery, where signals from the different cellular compartments converge and are integrated. Thus, they play a pivotal role in the amplification of the apoptotic processes initiated by DD receptors activation (p75 and Fas), down-regulation of growth factor signaling, mitochondrial influx of Ca^2+ ^released by ER and ROS accumulation in response to many brain insults. This function allows mitochondria to be the main *"sensor" *of the stress thresholds reached by neurons after an insult and continuously adjust their response. This role of mitochondria as *"sensor" *of the cellular conditions might be particularly relevant in AD and other chronic neurodegenerations, given that neurons, more than other cells, rather than dying, attempt to activate many protective programs that can delay the death decision, thus generating a situation of *"stand-by"*. This situation is exemplified in Figure 5 showing cells that accumulate under stress pressure following exposure to toxic stimuli and/or stressful events. The idea is that after the activation of a trigger event, a cell may activate in part the caspase pathway without reaching a fully activated state.

In this context, the activity of IAPs and Survivin, which can inhibit caspases, appears relevant, as well as their inhibition by Smac/DIABLO. As shown in Figure 4, IAPs can be induced by NF-kB and Akt, which can also inhibit the intrinsic apoptotic pathway through phosphorylation of both caspase-9 and its inactive form [150]. This modulation represents, indeed, another example of the fine regulatory control of apoptosis, and is consistent with the hypothesis that the final outcome (life or death) depends not only on which players are active in a given moment, as this decision also revolves around to what extent each pro-apoptotic and anti-apoptotic element is active, in terms of levels and persistency. Thus, it seems that the amplifications and brake mechanisms are meant to add a further level of complexity to the final steps controlling the life/death decisions that eventually will depend on the amount of mitochondrial pressure. As illustrated in Figure 5, cells will die when a "Point of No-Return" is reached. Beyond this checkpoint the anti-apoptotic mechanisms are no longer effective against a persistent condition of energy depletion following mitochondrial dysfunction. Thus, apoptosis would take place upon engulfment of an over-loaded homeostatic system.

Another element that has recently gained a lot of attention is the building up of evidences for cell cycle proteins and DNA tetraploidy in neurons in brain regions involved in AD degeneration, suggesting that neurons may enter aberrant cell cycle before undergoing apoptosis.

An appealing issue, as well as an interesting field for future directions, is the antiapoptotic activity of the Cki p27^Kip1 ^and p21^Cip1^. If, on the one hand, there is evidence indicating that cell cycle events are upstream of caspases activity [214], suggesting that they may function like a *"last-chance" *mechanism engaged by stressed cells to overcome and/or recover from endangering conditions, on the other hand, the role of Cki is interesting as potential inhibitors of apoptosis upon their translocation to cytoplasm followed by caspases-3/8-mediated cleavage [226] (Figure 4). The release of an active Cyclin/cdk complex would then explain the abortive onset of cell cycle.

However, the presence of cell cycle proteins has been detected also in the brain of rodents and humans under normal conditions, suggesting that processes linked to cell cycle events might have a physiological, rather than pathological role [230], and require further investigation. The presence of so many regulatory links yields complex and often counter-intuitive dynamics in response to changing conditions, therefore underlying the need for accurate modeling and extensive simulations to better understand the system and allow the development of a rational drug discovery process for neurodegenerative disorders.

In this context, there is a remarkable need to identify the check-points that represent the points of no-return for over-stressed cells. Given the existence of so many actors, it is clear the importance of deriving mathematical models that can allow us to identify these critical check-points, thereby allowing to focus on selected relevant pathways and speed up the discovery process of rate limiting steps useful for neuroprotection.

## Conclusion

In conclusion, this review has started from an overview of the current knowledge of the molecular events underlying apoptosis in neurodegenerative diseases, with particular attention to AD. Following the established strategy of modular systems biology, the data collected have been first used to identify the blueprint of the relevant modules of neuronal apoptosis and their wirings. Then, a molecular model of neuronal apoptosis has been constructed and discussed as a framework in which to investigate how various anti-apoptotic pathways may have a role in neuroprotection. This approach may offer new insights to the development of new therapeutic strategies against AD.

## Acknowledgements

This work was in part supported by Associazione Levi-Montalcini.

This article has been published as part of *BMC Neuroscience* Volume 7, Supplement 1, 2006: Problems and tools in the systems biology of the neuronal cell.  The full contents of the supplement are available online at .
